# Chest Wall Deformities in Adults With Fibrotic Interstitial Lung Disease Related to Surfactant-Related Gene Mutations

**DOI:** 10.1016/j.chpulm.2024.100106

**Published:** 2024-10-10

**Authors:** Margot Delin, Marie Pierre Debray, Marie Legendre, Lidwine Wemeau, Bruno Crestani, Emmanuel Brian, Justine Frija-Masson, Laurent Plantier, Spyros A. Papiris, Effrosyni D. Manali, Nadia Nathan, Raphael Borie

**Affiliations:** a Université Côte d'Azur, Centre Hospitalier Universitaire de Nice, IHU RespirERA, FHU OncoAge, Département de pneumologie et d’oncologie thoracique, Nice, France; b Service de Radiologie, Hôpital Bichat, APHP, and Université Paris Cité, Inserm, PHERE, Paris, France; c U.F. de Génétique moléculaire, APHP-Sorbonne Université; Laboratory of childhood genetic diseases UMR_S933, Inserm, Sorbonne Université; Hôpital Armand-Trousseau, Paris, France; d Service de pneumologie CHU Lille, Université de Lille, Lille, France; e Service de Pneumologie A Hôpital Bichat, APHP, Université Paris Cité, Inserm, PHERE, Paris, France; f Surgical department, Institut Mutualiste Montsouris, Paris, France; g Université Paris Cité, Inserm Neurodiderot, Assistance publique hôpitaux de Paris, Service de physiologie Explorations fonctionnelles, Hôpital Bichat Claude Bernard, Paris, France; h Service de pneumologie et explorations fonctionnelles respiratoires CHRU Tours, CEPR-Inserm UMR1100, Université de Tours, Tours, France; i 2nd Pulmonary Medicine Department, General University Hospital "Attikon", Medical School, National and Kapodistrian University of Athens; j Service de Pneumologie Pédiatrique et Centre de référence des maladies respiratoires rares RespiRare, APHP-Sorbonne Université; Laboratory of childhood genetic diseases UMR_S933, Inserm, Sorbonne Université; Hôpital Armand-Trousseau, Paris, France

To the Editor:

Chest wall deformity (CWD) is a structural abnormality reported in 0.4% of the general adult population.^1^ It can be acquired or congenital. Acquired CWD can occur due to chest surgery, radiotherapy, or hernia surgery,^2^ unlike congenital CWD that is diagnosed at birth.^3^ The most common types of CWD are pectus excavatum (88%, breastbone pushes inward), pectus carinatum (9%, breastbone and ribs protrude), and thoracic asymmetry.^3^ Scoliosis is overrepresented in patients with CWD (20%) compared with the general population (2%-4%).^4^ Most CWDs that appear before 3 years of age regress spontaneously; those occurring after 10 years of age deteriorate in one-third of cases.^5^ The potential association between chronic lung disease and increased incidence of CWD has been suggested with bronchopulmonary dysplasia and pectus excavatum < 1 year, or cystic fibrosis and pectus carinatum in childhood.^6^^,^^7^ However, the increased incidence of CWD in the context of fibrotic interstitial lung disease (FILD) has not been studied in adults.^8^

The following surfactant-related genes (SRGs) are implicated in FILD: *SFTPA1*, *SFTPA2*, *SFTPB*, *SFTPC*, *ABCA3*, and *NKX2-1*.^9^ Based on our personal observations of adult patients with FILD with *SFTPC* pathogenic variant who required a pectus excavatum surgery, our study aimed to describe CWD in adults with SRG-related FILD.

## Methods

We conducted a retrospective, observational study involving adult patients with interstitial lung disease (ILD) associated with a pathogenic variant in SRGs referred to Bichat, Paris, France, or Attikon, Athens, Greece, between 2005 and 2022. Patients were considered adults from 15 years of age because they can be referred to an adult physician in France. Inclusion criteria were as follows: (1) ≥ 15 years of age; (2) ILD on CT scan; and (3) pathogenic or probable pathogenic heterozygous variant in *SFTPA1*, *SFTPA2*, *SFTPC*, *NKX2-1*, or biallelic pathogenic variants in *ABCA3*, referred to as SRG mutations. Genetic analysis was offered according to French and European Respiratory Society^10^ recommendations to any patient with an idiopathic FILD before 50 years of age or with familial pulmonary fibrosis.

**Figure 1:**
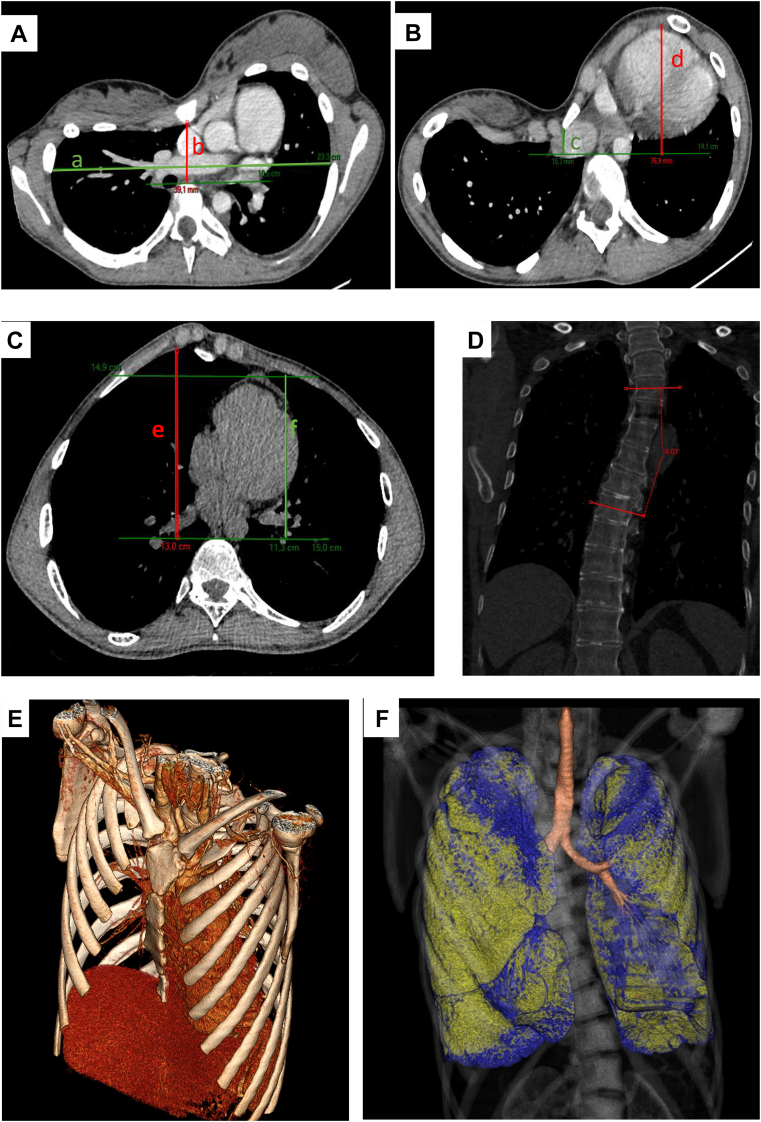
A-G, Pectus excavatum assessed by (A) Haller index of 5.2 and (B) correction index of 79% on chest CT scan. The Haller index is calculated by dividing the internal transverse diameter (a) by the internal anterior-posterior diameter (b) of the chest on the axial slice = (a)/(b). Pectus excavatum is defined by a Haller index > 2.5. The correction index quantifies the posterior displacement of the sternum relative to the ribs. Its measurements include the antero-posterior distance between the horizontal tangent to the anterior margin of the vertebral body and the posterior margin of the sternum or the most posterior depression of the adjacent costal cartilage (c) and the maximum distance from the horizontal tangent of the vertebral body to the inner margin of anterior chest cavity (d). Correction index = ((d) – (c))/(d). A correction index > 10% indicates pectus excavatum. (C) Illustration of fibrotic interstitial lung disease associated with pectus excavatum. Axial chest CT scan, lung window, showing ground-glass opacities with mild traction bronchiectasis in the left lower lobe. (D) Pectus carinatum assessed by correction index at 13% on chest CT scan. The correction index is still equal to ((e) – (f))/(e). Except that (e) is the antero-posterior distance between the horizontal tangent to the anterior margin of the vertebral body and the posterior margin of the sternum or the most anterior protrusion of the adjacent costal cartilage, and (f) is the distance between the horizontal tangent of the vertebral body and the line that follows the curvature of the thorax in the absence of pectus carinatum. A correction index > 10% defines a pectus carinatum. (E) Representative pectus excavatum with 3-dimensional reconstruction and (F) lung volume measurements of the same patient as in A and B. Total lung capacity was measured to be 3041 mL by plethysmography, whereas right lung volume was 1,266 mL and left lung volume was 918 mL by CT scan. –700 Hounsfield units (HU) was used as the cutoff for the segmentation of the normal lung: density < –700 HU was considered normal and is shown in green. A density > –700 HU was considered to be pathologic and colored in blue, which in this case mostly corresponded to ground glass opacities. (G) Cobb angle = 16.03°. The Cobb angle is measured between the two most inclined vertebrae. Cobb angle > 10° defines scoliosis.

The most recent CT scan was analyzed for the predominant ILD features, according to the Fleischner Society glossary.^11^ CWDs were the following: (1) pectus excavatum (Haller index > 2.5 and a correction index > 10%) (Fig 1A)^1^; (2) pectus carinatum: correction index > 10% (Fig 1B)^1^; and (3) thoracic asymmetry: volume difference of > 500 mL between the two lungs, resulting in a deviation of the mediastinum. Lung volume was calculated using CT lung density analysis on Vitrea (Canon Medical Informatics).

Scoliosis was defined by a Cobb angle on CT scan > 10° (Fig 1C).^12^

Each CT scan was analyzed independently by two investigators (M. P. D. and M. D.) at four different axial levels (T6 and T8 vertebrae, xyphoid and maximal deformation). For discordant results, a third analysis was made by both investigators together.

Data collected included age of diagnosis, weight, height, treatment, and pulmonary function test at the time of the CT scan.

All patients gave signed informed consent (CCTIRS no.08.015bis). Data are expressed as median (interquartile range) or number (%). Categorical and quantitative variables were compared by Fisher exact test and Wilcoxon rank sum test. Statistical significance was set at *P* < .05. All analyses were performed using GraphPad Prism 5.

## Results

Thirty-six adult patients with SRG mutations were referred, and nine were excluded (unavailable CT scan, n = 7; no ILD on CT scan, n = 2); all have been previously reported.^7^^,^^9^ Circumstances of SRG mutations diagnosis were childhood-onset FILD (< 15 years of age, n = 10, 36%), < 50-years-old adult-onset ILD (15-50 years of age, n = 13, 45%), and > 50-years-old adult-onset ILD but with a family pulmonary fibrosis (n =4, 14%) or after family screening (n = 1, 3%). None of them had CWD without FILD. The population was predominantly female (58%), with a median age of 39 years; 68% did not smoke (Table 1). None of the relatives who carried the variants without ILD (n = 11) had CWDs.

**Table 1:** Main Characteristics of the Patients Included and Comparisons of Lung Function Tests Depending on CWDs

Event	CWDs (n = 11)	Absence of CWDs (n = 17)	*P* Value
Demographic			
Male	5 (45)	7 (41)	>.99
Age at CT evaluation, y	32.5 (30.5-43.9)	45.3 (32.4-57.2)	.15
Age at first symptoms, y	19.5 (1.82-28.4)	34.9 (31.1-46.6)	**<.01**
Age at ILD diagnosis, y	19.5 (9.73-33.4)	37.1 (28.3-49.4)	**.024**
Active tobacco use	2 (18)	7 (41)	.25
Circumstances of diagnosis SRG mutations			
Childhood FILD^a^	6 (55)	4 (24)	.12
Adulthood FILD < 50 y	5 (45)	8 (47)	.93
Adulthood FILD ≥ 50 y + family history^b^	0 (0)	4 (24)	.13
Family screening	0 (0)	1 (5)	1
Surfactant-related gene implicated			
*ABCA3*	5 (45)	4 (24)	.24
*SFTPC*	4 (36)	3 (18)	NA
*SFTPA1* and *SFTPA2*	2 (18)	7 (41)	NA
*NKX2-1*	0 (0)	3 (18)	NA
Chest wall deformities			
Scoliosis	8 (72)	0 (0)	**< .001**
Cobb angle, °	11.6 (9.5-22.1)	4.6 (1.5-6.0)	**< .001**
Thoracic asymmetry	3 (27.2)	0 (0)	**< .001**
Pectus excavatum	4 (36.5)	0 (0)	**< .001**
Pectus carinatum	4 (36.5)	0 (0)	**< .001**
Maximal Haller index	3.1 (2.5-3.8)	2.2 (1.9-2.4)	**< .001**
Haller index at T6	2.9 (2.6-3.3)	2.2 (1.9-2.4)	**< .001**
Haller index at T8	2.6 (2.2-3.3)	2.0 (1.8-2.2)	**< .01**
Haller index at xyphoid	3.1 (2.3-3.6)	2.0 (1.8-2.2)	**< .001**
Maximal correction index, %	16.4 (14.3-29.2)	3.1 (0.0-3.9)	**< .001**
Correction index T6	10.7 (7.30-13.2)	2.7 (1.6-6.5)	**< .01**
Correction index T8	13.2 (11.4-16.4)	0.0 (0.0-4.0)	**< .001**
Correction index at xyphoid	15.4 (11.5-28.5)	3.1 (0.0-3.9)	**< .001**
CT scan signs			
Fibrosis (honeycombing, traction bronchiectasis, signs of distortion)	4 (36)	7 (41)	.89
Isolated ground glass opacities	3 (27)	6 (35)	NA
Ground glass opacities associated with cysts	3 (27)	3 (18)	NA
Isolated cysts	1 (9.1)	1 (5.9)	NA
Treatment ever received			
Corticosteroids	10 (91)	13 (76)	.62
Azithromycin	10 (91)	6 (35)	**< .01**
Hydroxychloroquine	6 (55)	7 (41)	.49
Antifibrotic	2 (18)	5 (29)	.67
Immunosuppressants	1 (9)	7 (41)	.099
Outcome			
Death	4 (36)	5 (31)	1
Age at death, y	34.4 (25.2-43.6)	47.9 (46.8-51.5)	.41
Eligible for transplantation (FVC < 50%)	8 (73)	5 (29)	.025
Lung transplantation	4 (36)	2 (12)	.19
Comparisons of lung function tests depending on CWDs			

	CWD+ (n = 11)	CWD− (n = 16)	Pectus excavatum (n = 4)	Pectus carinatum (n = 4)	*P* Value		
					CWD− vs CWD+	Excavatum vs CWD−	Carinatum vs CWD−
Height, cm	167 [8]	164 [9]	166 [6]	167 [9]	.399	.806	.686
Weight, kg	60 [17]	69 [22]	54 [11]	56 [16]	.270	.203	.289
FEV_1_, %	39 [13]	60 [18]	47 [9]	26 [8]	**.004**	.175	**.003**
FVC, %	41 [15]	61 [19]	45 [11]	32 [15]	**.007**	.125	**.013**
TLC, %	54 [22]	72 [25]	47 [12]	59 [26]	.086	.110	.417
FRC, %	77 [36]	86 [38]	71 [26]	96 [42]	.575	.534	.699
RV, %	109 [71]	93 [57]	61 [23]	171 [55]	.545	.358	**.041**
RV/TLC, %	222 [181]	125 [41]	128 [25]	357 [180]	.062	.888	**.002**
Dlco, %	32 [10]	49 [22]	37 [8]	24 [8]	**.038**	.373	.074

Data are mean [SD], No. (%), mean (interquartile range), or as otherwise indicated. CWD = chest wall deformity; Dlco = transfer capacity of the lung for carbon monoxide; FILD = fibrotic interstitial lung disease; FRC = functional residual capacity; ILD = interstitial lung disease; NA = not applicable; RV = residual volume; SRG = surfactant-related gene; TLC = total lung capacity. Values in bold font are statistically significant.

a All of these patients received long-term azithromycin.

b All of these patients carried an *SFTPA1* or *SFTPA2* variant.

Eleven patients (39%) presented with CWD: four with pectus excavatum,four with pectus carinatum, and three with thoracic asymmetries without any other CWD (Fig 1D). None of the patients had lobectomy or past medical history that could account for thoracic asymmetry. In addition, eight patients showed scoliosis, all with CWD: three with pectus excavatum, two with pectus carinatum, and three with thoracic asymmetry. The Cobb angle was significantly higher in the group with CWD (11.6°) than in the one without (4.6°) (*P* < .01) (Table 1).

Compared with patients without CWD, patients with CWD were younger, were diagnosed at a younger age, and received more frequently azithromycin (Table 1). Diagnosis was made at < 16 years of age in 5 of 11 patients (45%) with CWD vs 2 of 17 (11%) without CWD (*P* = .07). The median time between ILD and CWD diagnosis was 6.7 years (range, 0.0-17.6).

Patients with CWD had a more severe lung function with reduced FEV_1_ (39% vs 60%, *P* = .004), FVC (41% vs 61%, *P* = .007), and transfer capacity of the lung for carbon monoxide (32% vs 49%, *P* = .038). Residual volume was increased in both groups (Table 1).

## Discussion

We report that in this study, one-third of patients with FILD who were > 15 years of age with an SRG mutation showed CWD. To our knowledge, the association between adulthood FILD and CWD has never been described in the literature, except in pleuroparenchymal fibroelastosis and platythorax.^7^ CWD has been associated with different childhood diseases (eg, cystic fibrosis, bronchopulmonary dysplasia) but not with ILD. Whether the damage is related to ILD and to pulmonary fibrosis developed during childhood is specific to the SRG mutations, or a consequence of the treatment remains subject of hypotheses.

Several elements suggest that CWD could be related to the development of FILD in childhood, and to early fibrotic changes in the lung: a high elastic recoil due to ILD may have an impact on thorax and spinal bone growth over time. As SRGs are not expressed in bone tissue, it is unlikely that CWD is directly related to an SRG variant. As a comparison, the absence of CWD reported in 52 child-to-adult transition patients with sarcoidosis without pulmonary fibrosis highlights the probable impact of fibrosis on CWD.^13^ A younger age at symptoms onset and a younger age at diagnosis were associated with an increased prevalence of CWD. The respective responsibilities of the age at onset and the degree of fibrosis remain to be ascertained. The absence of traction of CWD (induced by fibrosis) in patients with bronchopulmonary dysplasia may explain the quasi absence of CWD in these pathologies in adulthood, whereas pleuroparenchymal fibroelastosis has been shown to be associated with platythorax.^7^ In this cohort, the age of onset of CWD was predominantly during teenage years or young adulthood. This is the age of the growth spurt, which could reveal or exacerbate any asymmetry in the rib/cartilage coupling.^14^ The association between CWD and scoliosis in this cohort is consistent with the reported 20% of pectus excavatum associated with scoliosis,^4^ suggesting that scoliosis could be a consequence of CWD rather than a consequence of the FILD.

The association between azithromycin and CWD may be attributed to a confounding factor, the age of onset, because azithromycin is frequently used in childhood ILD. The European task force for childhood ILD recommended the use of oral or IV steroids and an add-on therapy of either hydroxychloroquine or azithromycin with limited evidence.^15^ Those treatments may be considered in adults in addition to antifibrotic or immunosuppressants when indicated independently to the genetic status.^10^

This series has several limitations related to its retrospective design and the restricted number of available CT scans, justified by the patients’ age to limit their radiation exposure. It was also not possible to determine when CWD appeared to perform a multivariate analysis. We report a one-third prevalence of CWD in this cohort, but the actual prevalence of CWD in adult SRG mutations carriers needs to be clarified, considering the multiple biases of this cohort. Patients 15 to 17 years of age are not considered adults in several parts of the world, and the current cohort only reflects the experiences from Paris and Athens. This suggests that the prevalence of variants could be influenced by a founder effect specific to these regions.

Diagnosing CWD in patients with FILD with SRG mutation is of major importance. Patients with CWD have more severe lung testing and patients with pectus carinatum have the most severe volume reduction (residual volume, FVC, Table 1). ILD severity assessed by pulmonary function test may be overestimated. Seventy-three percent of patients with CWD showed FVC < 50% and were eligible for lung transplant, but only 27% underwent lung transplant. CWD could interfere with the realization of a lung transplantation and adaption of the lungs to the deformity and could have modified the indications for transplantation in this population.

We report a high frequency of CWD in young adults with FILD with SRG in Paris and Athens. We hypothesize that early ILD onset could lead to a constrained lung growth with subsequent thoracic CWD.

## Financial/Nonfinancial Disclosures

The authors have reported to *CHEST Pulmonary* the following: N. N. has received grants or contracts (CORTICONEHI: Clinical trial, Million Dollar Bike Ride, Chancellerie des Universités, Respifil) and payment for articles (La lettre du pneumologue). J. F.-M. has received grants or contracts from LVL medical, has received payment for presentations from SOS oxygène, and has received assistance for support for attending meetings from Vitalair, SOS oxygène, LVL medical, and ADEP. M. P. D. has received payment for presentations and support for attending meetings from Sanofi and Boehringer-Ingelheim. B. C. has received payment for presentations, monitoring boards, and support for attending meetings from Astra Zeneca, BMS, Boehringer Ingelheim, GSK, Novartis, Roche, and Sanofi; and is the president of the board of trustees of the Fondation du Souffle (a French charity). R. B. has received payment for presentations and support for attending meetings from Sanofi, Boehringer-Ingelheim, and Ferrer. S. A. P. has received honoraria for lectures from Boehringer Ingelheim, ELPEN Hellas, Hoffman La Roche, and DEMO Hellas; and support for attending meetings and/or travel from Boehringer Ingelheim and ELPEN Hellas. E. D. M. has received honoraria for lectures from Boehringer Ingelheim, Astra Zeneca, ELPEN Hellas, Hoffman La Roche, and DEMO Helllas; and support for attending meetings and/or travel from Boehringer Ingelheim and ELPEN Hellas. None declared (M. D., L. W., M. L., L. P., E. B.).
